# Non-centrosomal TPX2-Dependent Regulation of the Aurora A Kinase: Functional Implications for Healthy and Pathological Cell Division

**DOI:** 10.3389/fonc.2016.00088

**Published:** 2016-04-15

**Authors:** Georgina Garrido, Isabelle Vernos

**Affiliations:** ^1^Cell and Developmental Biology Programme, Centre for Genomic Regulation (CRG), Barcelona Institute of Science and Technology, Barcelona, Spain; ^2^Universitat Pompeu Fabra (UPF), Barcelona, Spain; ^3^Institució Catalana de Recerca I Estudis Avançats (ICREA), Barcelona, Spain

**Keywords:** Aurora A kinase, TPX2, spindle, RanGTP, microtubule, cell division, importin, phosphorylation

## Abstract

Aurora A has been extensively characterized as a centrosomal kinase with essential functions during cell division including centrosome maturation and separation and spindle assembly. However, Aurora A localization is not restricted to the centrosomes and compelling evidence support the existence of specific mechanisms of activation and functions for non-centrosomal Aurora A in the dividing cell. It has been now well established that spindle assembly involves an acentrosomal RanGTP-dependent pathway that triggers microtubule assembly and organization in the proximity of the chromosomes whether centrosomes are present or not. The mechanism involves the regulation of a number of NLS-containing proteins, generically called SAFS (Spindle Assembly Factors) that exert their functions upon release from karyopherins by RanGTP. One of them, the nuclear protein TPX2 interacts with and activates Aurora A upon release from importins by RanGTP. This basic mechanism triggers the activation of Aurora A in the proximity of the chromosomes potentially translating the RanGTP signaling gradient centered on the chromosome into an Aurora A phosphorylation network. Here, we will review our current knowledge on the RanGTP-dependent TPX2 activation of Aurora A away from centrosomes: from the mechanism of activation and its functional consequences on the kinase stability and regulation to its roles in spindle assembly and cell division. We will then focus on the substrates of the TPX2-activated Aurora A having a role in microtubule nucleation, stabilization, and organization. Finally, we will briefly discuss the implications of the use of Aurora A inhibitors in anti-tumor therapies in the light of its functional interaction with TPX2.

Cell cycle progression is crucial for cell viability. During mitosis, most cellular components undergo a dramatic reorganization. In particular, the relatively stable interphase microtubule (MT) network disappears and highly dynamic MTs organize the bipolar spindle, the molecular machine that provides the support and forces for chromosome segregation. The progression and coordination of the events that drive spindle assembly and culminate with the birth of two daughter cells rely on complex regulatory networks involving several kinases. One of them is Aurora A (1), a kinase originally identified in *Drosophila* (2). In higher organisms, Aurora A is a member of the Aurora kinase family consisting of three serine–threonine kinases whose expression and kinase activity peak in M phase (Figure 1). Aurora kinases have essential roles during cell division and in particular in centrosome duplication and separation, spindle assembly, chromosome alignment, spindle assembly checkpoint, central spindle assembly, and cytokinesis (3–5).

**Figure 1:**
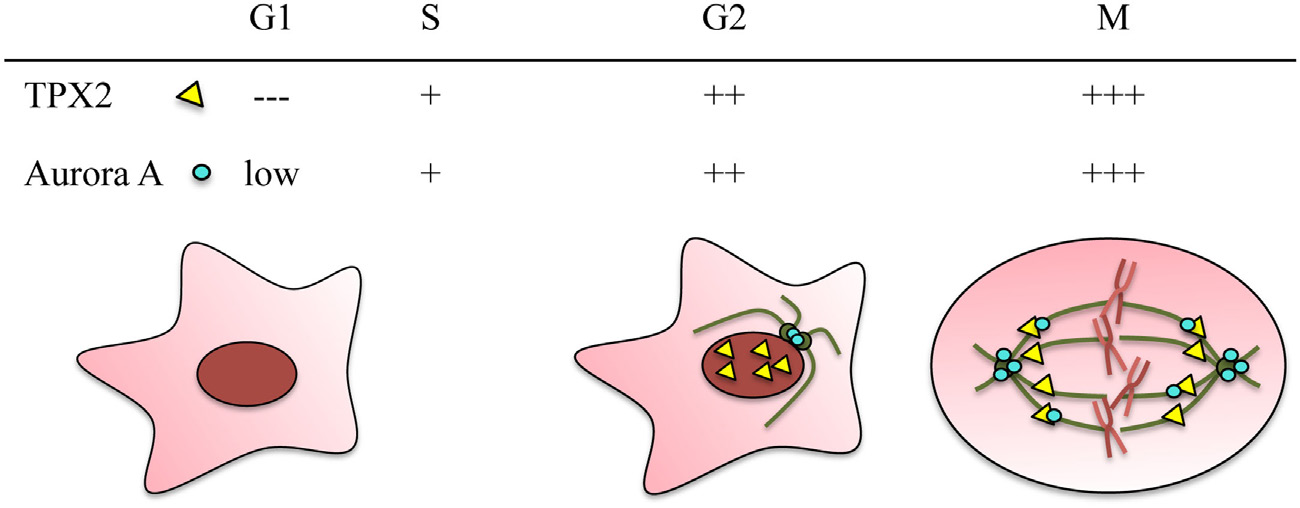
**TPX2 and Aurora A are cell cycle regulated proteins**. Both proteins accumulate during G2/M and are degraded through the APC/C proteasome pathway at the end of mitosis. The relative protein levels of TPX2 (yellow triangle) and Aurora A (blue circle) in the different cell cycle phases are represented at the top. The localization of the two proteins during these cell cycle phases is represented in the drawings. In G2, TPX2 accumulates inside the nucleus whereas Aurora A accumulates at the centrosomes. During mitosis, both proteins co-localize along the spindle microtubules, and Aurora A also accumulates at the centrosomes.

The potential link between the Aurora kinases and tumor initiation and/or development has fueled the interest in understanding their function and regulation over the last years. Indeed, Aurora A gene is located in a region of chromosome 20 that is frequently overexpressed in human cancers (6, 7), and it is found in higher levels in many tumor types (8–10). Moreover, it shows oncogenic properties (3, 11, 12). Aurora A gene is also a candidate low penetrance cancer-susceptibility gene (13, 14). Aurora A is therefore considered as a potentially useful molecular therapeutic target, and several specific small molecule inhibitors are currently being tested in clinical trials (15–18).

Although the three Aurora kinases share a conserved catalytic domain, a few critical amino acid substitutions in their catalytic domains confer activator specificity. Moreover, divergent N- and C-terminal domains provide specificity at least in part through protein–protein interactions and distinct subcellular localizations during mitosis. While Aurora B and C localize to the kinetochores and the anaphase central spindle as part of the chromosomal passenger complex (19), Aurora A localizes to the centrosome throughout cell division and is often described as a centrosomal kinase (Figure 1) (20). However, Aurora A also localizes along the spindle MTs and performs essential functions unrelated to its centrosomal localization. Here, we will focus on the TPX2-dependent regulation and function of acentrosomal Aurora A during cell division.

## Aurora a Kinase Activation

The activity of Aurora A is regulated by phosphorylation–­dephosphorylation (21, 22). In particular, the autophosphorylation of Thr288 (in humans), a residue residing within the activation loop of the catalytic domain, has been described as critical for kinase activity (11). In addition, other kinases may phosphorylate Thr288, and *in vitro* assays showed that PKA phosphorylates Aurora A on at least three residues, including Thr288 (11, 22). Specific anti-Phosho-Thr288 antibodies have been useful to monitor when and where Aurora A is active in tissue culture cells, revealing that the kinase is activated at the centrosomes and the spindle microtubules proximal to the poles during prometaphase and metaphase (23). However, some controversy regarding Aurora A activation has recently emerged because phosphorylation on Thr288 alone was shown to be insufficient for the kinase to adopt a fully active conformation (24). On the other hand, there is evidence that activation may occur in the absence of Thr288 phosphorylation (see below).

**Figure 2:**
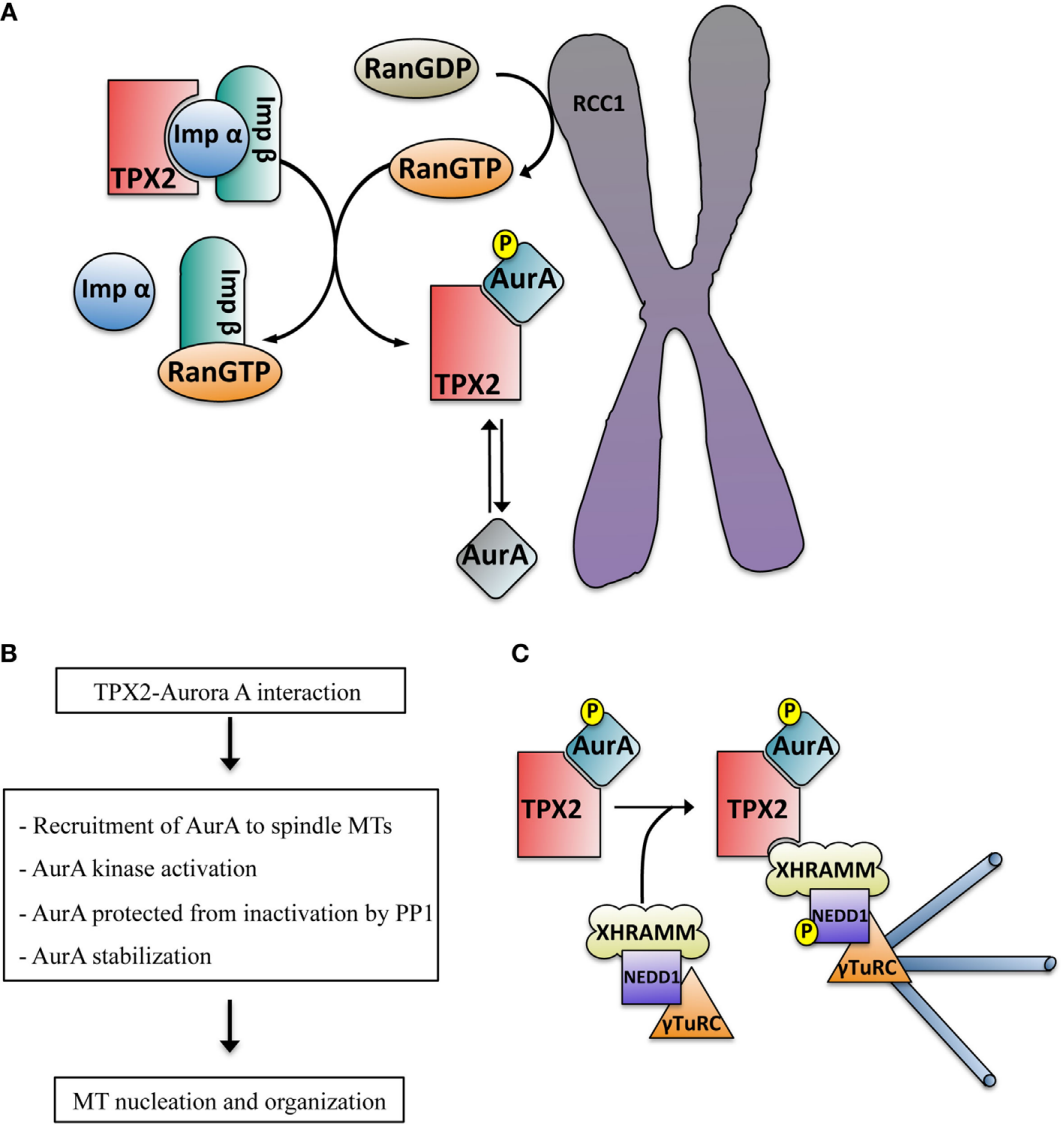
**(A)** Schematic representation of the RanGTP-dependent interaction between TPX2 and Aurora A in the proximity of the chromosomes. The exchange factor RCC1 associated with the chromosomes generates a peak of RanGTP that releases TPX2 from the importin alpha and beta complex. TPX2 can then bind to Aurora A, promoting its autophosphorylation on Thr288 and kinase activation (blue color). The phosphatase PP1 can inactivate TPX2 free active Aurora A (mainly at the centrosome, gray color) through dephosphorylation but not the TPX2-activated Aurora A (blue color). **(B)** Direct consequences of the TPX2–Aurora A interaction. **(C)** Schematic representation of the mechanism driving acentrosomal RanGTP MT nucleation triggered by the complex TPX2–Aurora A. The TPX2–Aurora A complex associates with another specific complex containing XRHAMM-NEDD1–γ-TurC. In this macro complex the activated Aurora A phosphorylates NEDD1 at Ser405, an essential prerequisite for MT nucleation.

Aurora A activation can also be triggered through allosteric interactions with a number of proteins such as Ajuba, Bora, protein phosphatase inhibitor-2, nucleophosmin, and PAK (25–29). A specific mechanism drives Aurora A activation in a RanGTP-dependent manner in dividing cells (21, 30, 31) (Figure 2A). This mechanism involves TPX2, a cell cycle regulated nuclear protein essential for chromosome and RanGTP-dependent MT nucleation (32, 33) and bipolar spindle assembly whether centrosomes are present or not (34–36) (Figure 1). TPX2 release from importins is triggered by RanGTP in the proximity of the chromosomes and enables its interaction with Aurora A thereby promoting its local activation in a centrosome-independent manner (34).

## Consequences of TPX2 Interaction with Aurora A

The interaction between TPX2 and Aurora A has several important functional consequences including the targeting of Aurora A to the spindle microtubules (37) and the assembly of spindles of the correct length that faithfully segregate chromosomes (38). Mechanistically it drives the activation of Aurora A (21, 30) through a direct interaction between the catalytic domain of Aurora A and the first 43 residues of TPX2 in humans (39 residues in *Xenopus*) (30, 37). Despite the high degree of conservation between the catalytic domains of Aurora A and B, the interaction between Aurora A and TPX2 is highly specific. Indeed, a single amino acid difference in the catalytic domain of Aurora B is sufficient to impair its interaction with TPX2 (39).

Structural studies showed that the binding of TPX2 to Aurora A promotes a conformational change in its catalytic domain involving the reorganization of the activation segment, providing a good binding platform for substrates (30). This also triggers Aurora A autophosphorylation at Thr288 in human cells (Thr295 in *Xenopus laevis*) (21) contributing to its activation (Figure 2A). Although it has been shown that TPX2 can fully activate Aurora A in the absence of Thr288 phosphorylation (40), other authors have proposed that Aurora A Thr288 phosphorylation and TPX2 binding act synergistically for the full kinase activation (41).

The conformational change induced by the binding of TPX2 to Aurora A results in the change in position of Thr288 that moves it into a buried position inaccessible to inactivating phosphatases (30). Therefore, TPX2 not only activates Aurora A but it “locks” the kinase into an active conformation that cannot be readily inactivated by PP1 like TPX2 free Aurora A (Figure 2A). Interestingly, the phosphatase PP6 was recently shown to specifically target the Aurora A–TPX2 complex triggering the dephosphorylation of the protected Thr288 thereby regulating Aurora A activity and consequently, spindle formation (42).

Finally, TPX2 protects Aurora A from degradation that occurs under normal conditions at the end of mitosis through the cdh1 activated APC/C proteasome pathway (43). Certainly, TPX2 depletion promotes a premature decrease of Aurora A levels in prometaphase (44) (Figure 2B).

Other functional implications of the TPX2–Aurora A interaction may derive from the phosphorylation of TPX2 itself. Indeed, TPX2 is a substrate of Aurora A. *Xenopus* Aurora A phosphorylates TPX2 on three serine residues (Ser48, Ser90, and Ser94) (21, 45). In HeLa cells Aurora A phosphorylated TPX2 was shown to control mitotic spindle length (46). However, the specific function of the Aurora A-dependent phosphorylation of TPX2 is still not entirely clear. In addition, TPX2 phosphorylation by the essential mitotic kinase polo-like kinase 1 (Plk1) was reported to increase its ability to activate Aurora A (47) while ckd1/2-dependent TPX2 phosphorylation was shown to regulate TPX2 localization impacting spindle assembly via Aurora A and Eg5 (48).

## Functional Relevance of TPX2-Dependent Aurora A Phosphorylation During Mitosis

TPX2 and Aurora A both perform essential functions during cell division although not all of them are dependent on their interaction. Aurora A null mouse embryos, similar to TPX2 ablation, are embryonic lethal failing to undergo the morula-blastocyst transition due to defects in mitosis (49–51).

The functional consequences of Aurora A activation by TPX2 in the dividing cell have to be examined in the context of the function and regulation of TPX2 during cell division. In *Xenopus* egg extract and in mammalian cells TPX2 is essential for acentrosomal MT assembly driven by the chromosome-dependent RanGTP pathway in M phase (33, 52). In turn, this pathway is essential for the assembly of a functional spindle that can drive faithful chromosome segregation to the daughter cells (35, 53–55).

Ran cycles between an inactive GDP-bound state and an active GTP-bound state, which is controlled by regulatory proteins. The Ran exchange factor RCC1 localizes to the mitotic chromosomes whereas other factors that promote RanGTPase activity (RanGAP1 and RanBP1) are cytosolic. This promotes the formation of a RanGTP gradient centered on the chromosomes that has been directly visualized in *Xenopus* egg extracts (56–58) and in mammalian cells (59, 60). In the dividing cell, RanGTP provides a spatial signal that triggers MT assembly in the proximity of the chromosomes and their organization into a bipolar spindle [reviewed in Ref. (61)]. In mammalian cells the system may however be more complex since it has been shown that components of the Ran system, including RanGTP, localize to the centrosome and play an important role in MT nucleation (62–64). One essential target of the RanGTP pathway away from the centrosome is the nuclear protein TPX2. Work performed in *Xenopus* egg extracts showed that RanGTP promotes the dissociation of TPX2 from inhibitory interactions with importin-α/β in the vicinity of chromosomes (52, 65). This release enables the interaction of TPX2 with Aurora A leading to its activation. Therefore, it is tempting to speculate that the RanGTP gradient translates into an Aurora A-dependent phosphorylation signaling network.

Some functional implications of the TPX2-dependent interaction with and activation of Aurora A have been recently uncovered through the characterization of the mechanism underlying RanGTP-dependent acentrosomal MT nucleation in *Xenopus* egg extract (32). In higher eukaryotes MT nucleation is driven by the γ-tubulin ring complex (γ-TuRC), a multi-subunit complex consisting of multiple copies of γ-tubulin and a number of associated proteins named as gamma-tubulin complex proteins (GCPs) (66, 67). Together with the adaptor protein NEDD1, γ-TuRC is required for all the MT nucleation pathways described in mitosis (68, 69). Another specific requirement for the RanGTP pathway is TPX2 (65). Recently, we showed that RanGTP promotes the association of TPX2 with a XRHAMM-NEDD1– γ-TuRC complex that includes Aurora A. We also showed that within this complex the TPX2-activated Aurora A phosphorylates NEDD1 on Ser405 an essential step for RanGTP-dependent MT nucleation (32, 70) (Figure 2C).

Another RanGTP-dependent protein complex containing TPX2 and Aurora A was previously identified in *Xenopus* egg extract (71) and shown to be required for RanGTP-dependent MT organization. This complex includes the tetramic plus-end directed motor Eg5, XMAP215 and the RanGTP target HURP. In *Xenopus* egg extract and in mammalian cells, TPX2 regulates Eg5 activity through a direct interaction (72, 73). Although Aurora A phosphorylates Eg5 (74) no function for this phosphorylation in spindle formation was identified in *Xenopus* egg extracts (75). On the other hand, HURP is necessary for K-fiber stabilization in mammalian cells (76–78) and its phosphorylation by Aurora A is required for MT binding (79). Altogether these data suggest that some proteins may be specific substrates of the TPX2-Aurora A complex. However, further work is needed to test this idea.

The dual role of TPX2 in activating and localizing Aurora A to the spindle microtubules through an allosteric interaction is not unique. For example, besides the classical activation of the MAPK p38α by MAPKK, p38α can be activated by TAB1 [transforming growth factor-β-activated protein kinase 1 (TAK1)-binding protein 1] as well (80). The binding of TAB1 to p38α promotes its autophosphorylation and consequently, its activation. Concerning the targeting role, a similar mechanism is at play for the A-kinase anchoring proteins (AKAPs). AKAPs bind directly PKA and recruit it to specific subcellular localizations where the kinase activity is required (81). AKAPs also function as scaffold proteins to facilitate the formation of multiprotein complexes. TPX2 may also provide a scaffolding activity. It may have a critical role for the recruitment of the MT nucleation complex and NEDD1 phosphorylation by Aurora A. Similarly, it may also act as a scaffold for the HURP containing complex whose formation and function depends on Aurora A activity, and consequently on TPX2 (71).

## Conservation of the TPX2–Aurora A Module?

Aurora kinases are found in a wide range of organisms from yeast to humans and they have conserved functions during cell division. TPX2 orthologs have also been identified in a variety of genera and different kingdoms. Interestingly, the *tpx2* knockout mice display severe developmental defects and embryonic lethality (82) and similar phenotypes were described for a *tpx2* knockout in *Arabidopsis thaliana* (83).

Aurora A was identified in *Drosophila*. However, it is only recently that Ssp1/Mei-38 was proposed to be a putative TPX2 ortholog (84). Although the effects of loss-of-function of this protein are less severe than in the case of human TPX2, Ssp1/Mei-38 shows similar localization to spindle microtubules. Moreover, it also contains a sequence conserved in human TPX2 that confers the microtubule-binding and bundling activities. However, Ssp1/Mei-38 lacks an Aurora A binding domain suggesting that it does not fulfill the same role as the vertebrate TPX2 during cell division and therefore it may not be a true TPX2 ortholog.

*Caenorhabditis elegans* has two Aurora-like kinases. A putative ortholog of TPX2 was recently identified and named TPX2-like protein (TPXL-1) (85). Although TPXL-1 activates and localizes Aurora A to the mitotic spindle and not to the centrosome, it actually does not share other essential features and functions of TPX2 like its RanGTP regulation and its role in microtubule nucleation (86).

These data suggest that different evolutionary modules may exist to control the localization and activation of the Aurora kinases during cell division. Vertebrates seem to have developed a unique module to integrate the control of localization and activation of Aurora A by the chromosomal RanGTP-dependent pathway through a single interacting protein, TPX2.

## Cancer and Therapeutics: TPX2 and Aurora are Overexpressed in Different Tumors

Aurora A and TPX2 are overexpressed in several types of tumors and have been implicated at different levels in cancer. Although the mechanism underlying the role of TPX2 and Aurora A in tumorigenesis may be at least in part independent, there are data to suggest a role for the complex. TPX2 was initially identified as a proliferation marker with a potential role in human cancer (87). It is indeed overexpressed in many tumor types (88). High levels of Aurora A were detected in many cancer types including prostate cancer, gastric carcinoma, breast carcinoma (89), ovarian cancer, laryngeal carcinoma, bladder cancer, and pancreatic carcinoma, among others (90). Moreover, both genes are part of the chromosomal instability signature that was found to predict clinical outcome for different cancers with TPX2 having the highest CIN score (91).

Interestingly, both TPX2 and Aurora A genes are located on chromosome 20q, whose amplification is found in tumors and moreover, co-expression of TPX2 and Aurora A has been observed in some tumors (92). For instance, Aurora A and TPX2 were found overexpressed in lung cancer cells (93), different colon cancers (94, 95) and neuroblastoma (96). Based on the correlation of co-expression it was in fact proposed that TPX2 and Aurora A might act as a functional unit (90). Interestingly, a mutant of Aurora A (S155R), that is unable to interact with TPX2, has been identified in colon cancer (97), suggesting that the misregulation of Aurora A localization and/or activity may also be deleterious for the cell. It is also interesting to note in this context that the tumor suppressor p53 is regulated by both TPX2 and Aurora A in *Xenopus* (98).

Some data suggest that the increased levels of Aurora A in various tumors may be the consequence of protein stabilization rather than gene amplification. Indeed, phosphorylation of Aurora A Ser51 inhibits its degradation via the cdh1 activated ubiquitin ligase APC/C at the end of mitosis and Aurora A constitutively phosphorylated at Ser51 was shown to be present in neck and head cancer tissues with Aurora A overexpression (99). Although no direct connection has been reported yet, it is interesting to note here that TPX2 protects Aurora A from degradation potentially contributing to the maintenance of high levels.

The clear implications of Aurora A in cancer have promoted the intensive search for small molecule inhibitors for their potential therapeutic use (100). Some of them already show interesting potential in clinical trials but a further optimization may be required. Targeting specifically the TPX2-activated Aurora A may open new strategies in cancer therapy.

## Author Contributions

All authors listed, have made substantial, direct, and intellectual contribution to the work, and approved it for publication.

## Conflict of Interest Statement

The authors declare that the research was conducted in the absence of any commercial or financial relationships that could be construed as a potential conflict of interest.

## Abbreviations

γTuRC, γ-tubulin ring complex; APC/C, anaphase-promoting complex; K-Fiber, kinetochore-fiber; MAP, microtubule-associated protein; MT, microtubule; NLS, nuclear localization signal; SAF, spindle assembly factor.
